# Human Cytomegalovirus and Risk of Incident Cardiovascular Disease in UK Biobank

**DOI:** 10.1093/infdis/jiab571

**Published:** 2021-11-23

**Authors:** Tom A Yates, Gareth J Griffith, Tim T Morris

**Affiliations:** 1 Department of Infectious Disease, Faculty of Medicine, Imperial College London, London, United Kingdom; 2 Medical Research Council Integrative Epidemiology Unit at the University of Bristol, Bristol, United Kingdom; 3 Population Health Sciences, Bristol Medical School, University of Bristol, Bristol, United Kingdom

To The Editor—Hamilton et al’s [1] analysis of the association between cytomegalovirus (CMV) infection and incident cardiovascular disease (CVD) in 8531 UK Biobank (UKBB) participants is stronger than most observational studies of the CMV-CVD association. There was careful adjustment for age, sex, socioeconomic position, and cardiovascular risk factors. Neither inflammatory markers nor prior CVD were included as covariates, which would have been inappropriate. However, although the authors acknowledge that sections of the population are underrepresented in the UKBB, the potential impact of this on their main results is not discussed.

Nonrandom sample selection can lead to spurious in-sample associations, a form of collider bias. A schematic example is presented in Figure 1A, in which individuals with high CVD risk or high CMV exposure are less likely to be sampled. This induces a negative correlation between CVD and CMV in the sample that does not hold in the broader population. To evaluate the extent to which collider bias might explain Hamilton et al’s [1] null findings, we compared CVD risk and CMV exposure in UKBB participants to that in more representative population samples.

**Figure 1.**
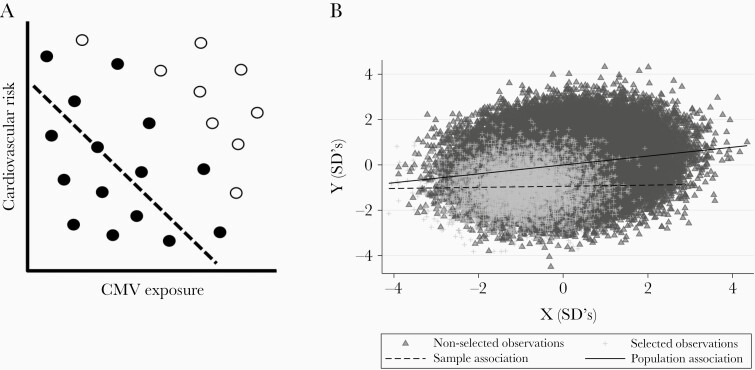
(A) Schematic demonstrating an in-sample negative correlation (filled circles and dashed line) that does not exist in the underlying population (all circles) may be generated by selectively including individuals with lower levels of both exposure and outcome. (B) Simulated data showing how in-sample associations (dashed line) may not reflect population level associations (line) if selected individuals (crosses) are a specific subset of the population (triangles).

UKBB is a highly selected sample. Of 9 million 40- to 69-year-olds invited to participate, only 5.5% enrolled [2]. The sample is enriched for individuals of higher socioeconomic position and white ethnicities (94.6% vs 91.3%) [2]. UKBB participants are less likely to be obese, drink less alcohol, and are less likely to smoke compared with more population representative respondents to the Health Survey for England (HSE) [2]. All-cause mortality rates among UKBB participants aged 70–74 years are 46% and 56% lower in men and women, respectively, than in the general population of England and Wales [2].

UKBB participants have lower self-reported CVD prevalence at enrollment [2], lower exposure to CVD risk factors [2, 3], and CVD mortality rates that are 2.9-fold lower than individuals of a comparable age in the HSE [3]. Previous analyses have shown that these selection effects can attenuate risk factor-CVD associations. For example, for hypertension, the hazard ratio for CVD mortality is 1.9 in UKBB and 2.6 in HSE [3].

Because CMV is more prevalent in those of lower socioeconomic position [4, 5], it is plausible that UKBB participants have lower CMV exposure than the general population. Cytomegalovirus seroprevalence appears lower in UKBB than in participants from a 2002 serosurvey using blood samples from individuals accessing National Health Service (NHS) care in England and Wales [6]. Age-specific CMV seroprevalence in the serosurvey was only presented graphically, preventing quantification of these differences.

Comparing the age-specific CMV seroprevalence reported by Hamilton et al [1] with that among pregnant women in the Born in Bradford (BiB) birth cohort [7] suggests markedly lower CMV prevalence in UKBB. Cytomegalovirus seropositivity is more prevalent in older individuals, which is partly a cohort effect, with most incident infections occurring in individuals under the age of 40 years [6]. Seroprevalence in 40- to 49-year-olds in UKBB is 48.0%, similar to that among pregnant white British women in BiB (48.6%), despite the latter having a mean age of 26.6 years [1, 7]. Cytomegalovirus is also more prevalent in people of non-white ethnicity [4, 5, 7]. Seroprevalence among United Kingdom-born pregnant women of South Asian ethnicity in BiB was 89.3% (mean age 27.4 years). Note that CMV seroprevalence in UKBB is similar in men (56.0%) and women (59.3%) [1].

It appears that UKBB participants have lower CVD and CMV prevalence than the general population. The potential impact of selection effects into UKBB on observed associations can be simulated. For example, selecting 5% of individuals with lower risk of both characteristics can produce a null in-sample association if the 2 characteristics have a Pearson’s correlation coefficient of only 0.2 in the broader population (Figure 1B). Here, both characteristics are treated as continuous variables, eg, CVD incidence and lifetime exposure to CMV. The code to reproduce this analysis under varying selection mechanisms is available at https://github.com/timtmorris/collider-bias.

Collider bias is a common and underappreciated problem in health research. Its potential to impact analyses in UKBB and other unrepresentative samples is well described [3, 8, 9]. This bias may be particularly consequential when investigating the impact of ubiquitous exposures on common diseases. Here, modest differences in attributable risk imply a substantial burden of disease.

The best solution to this problem would be to invest in making participation in research accessible to a broader section of the community. This includes access to healthcare services that routinely contribute data to, eg, Hospital Episodes Statistics. Where this has not been done, researchers should conduct their analyses in other less selected datasets or, where data exist on the determinants of inclusion, weight the sample and adjust for selection. This is an area of active methodological research [10].

## Notes

***Financial support*.** T. A. Y. is funded via a National Institute for Health Research (NIHR) Academic Clinical Fellowship (ACF-2018-21-007) and acknowledges support from the NIHR Imperial Biomedical Research Centre. The Medical Research Council (MRC) and the University of Bristol support the MRC Integrative Epidemiology Unit (MC_UU_00011/1, MC_UU_00011/3).

***Potential conflicts of interest*.** T. A. Y. is currently applying for funding to undertake research on the indirect consequences of cytomegalovirus infection. All authors have submitted the ICMJE Form for Disclosure of Potential Conflicts of Interest. Conflicts that the editors consider relevant to the content of the manuscript have been disclosed.
